# Supplementary material for: A coronene-based semiconducting two-dimensional metal-organic framework with ferromagnetic behavior
Source: Nat Commun. 2018 Jul 6;9:2637. doi: 10.1038/s41467-018-05141-4 (PMC6035257; doi:10.1038/s41467-018-05141-4)
Supplement: Supplementary file 1 — Supplementary Information [file 41467_2018_5141_MOESM1_ESM.pdf]

A Coronene-Based Semiconducting Two-Dimensional Metal-Organic Framework with Ferromagnetic Behavior

Renhao Dong et al.

Supplementary Figures

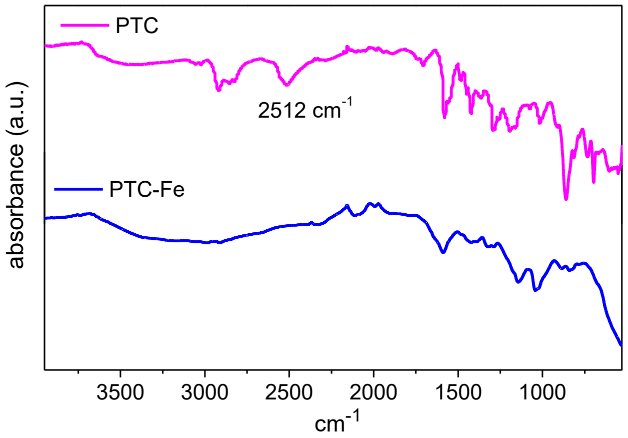

Supplementary Figure 1 | FT-IR analysis of the PTC-Fe 2D MOF. The attenuated total reflection IR (ATR-IR) spectra of the PTC-Fe and PTC monomer are compared. Whereas the PTC monomer exhibited a strong signal at 2512 cm^{-1} attributable to the S-H stretching vibrations, this peak vanished in the PTC-Fe, suggesting that the thiol groups were efficiently coordinated to Fe ions to form iron bis(dithiolene) linkers.

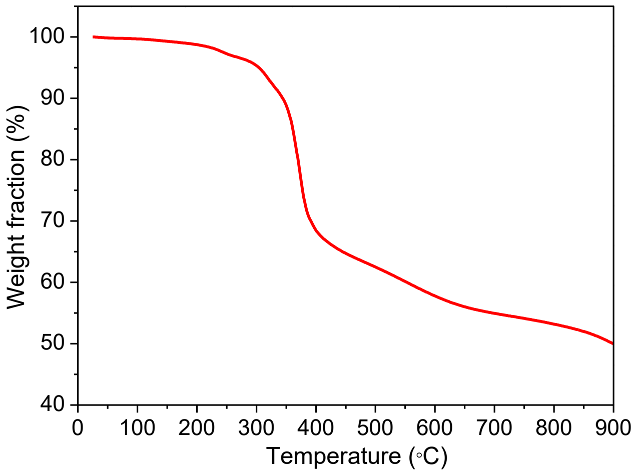

Supplementary Figure 2 | TGA spectrum of PTC-Fe 2D MOF measured under nitrogen atmosphere.

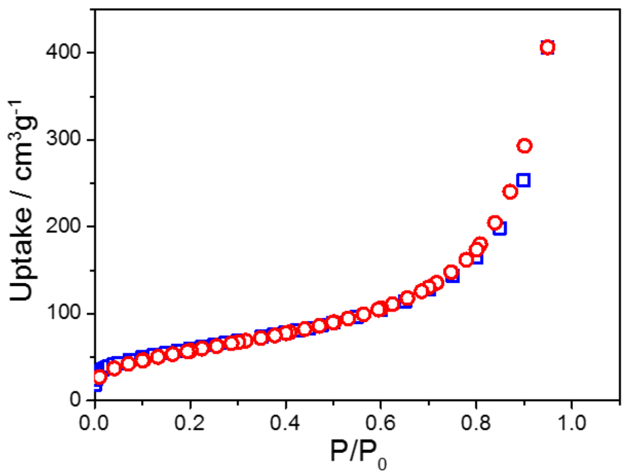

Supplementary Figure 3 | N_2 sorption isotherms of PTC-Fe 2D MOFs at 77 K reveal a Brunauer-Emmett-Teller surface area of 210(±5) m^2 g^{-1}. Black dots: adsorption. Red dots: desorption. The counter ions of NH_4^+ took up the pores in PTC-Fe MOF, leading to the relatively low BET surface area.

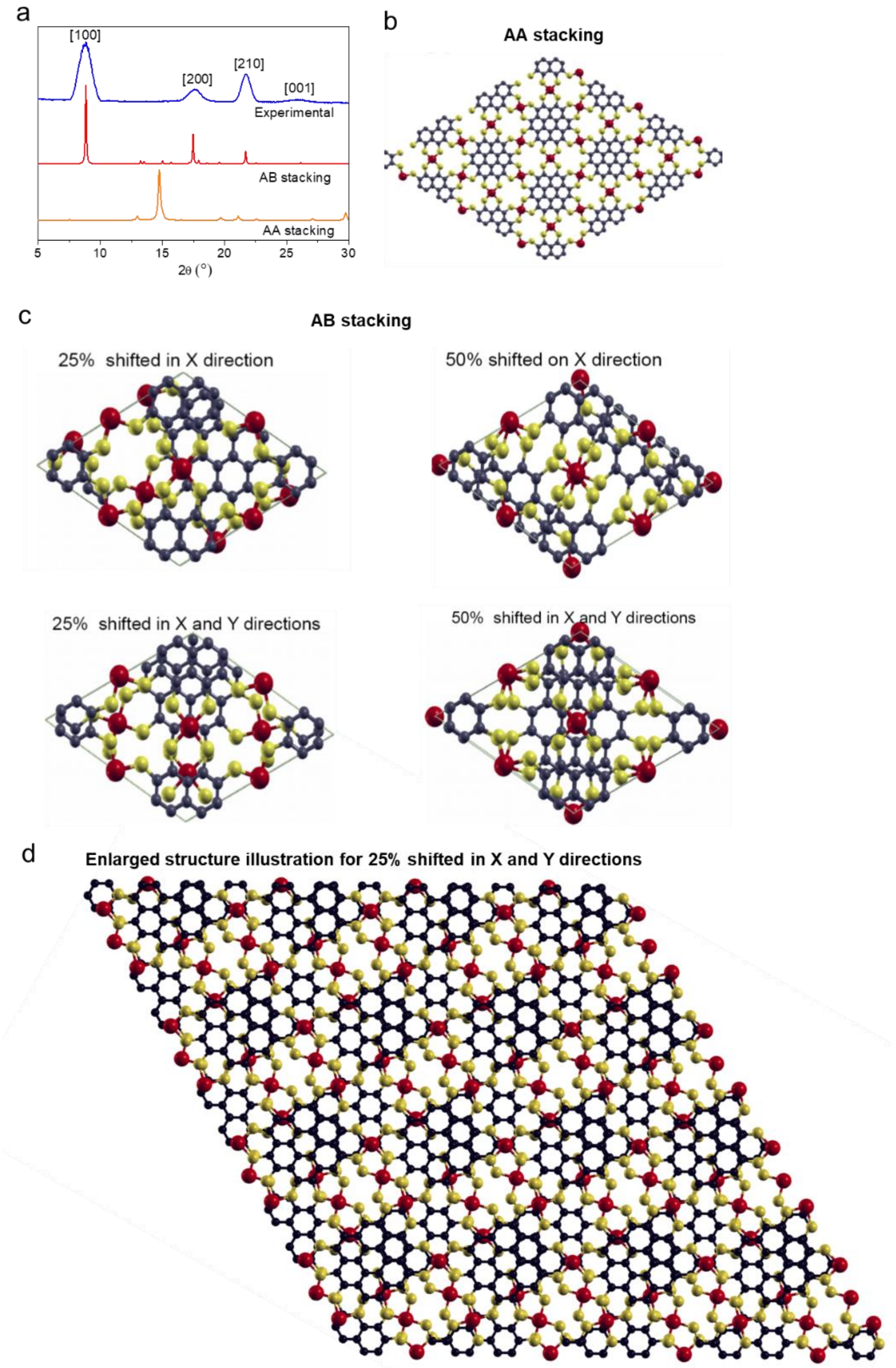

Supplementary Figure 4 | Crystal structure analysis of the PTC-Fe 2D MOF by powder XRD. a, experimental and simulated PXRD patterns. **b**, simulated AA-stacking arrangements for the layers of PTC-Fe. **c**, various AB tacking models. After comparison, the PTC-Fe 2D MOFs are determined to present AB stacking model with 25% shifting in X and Y directions. **d**, the enlarged structure illustration for AB stacking with 25% shifting in X and Y directions.

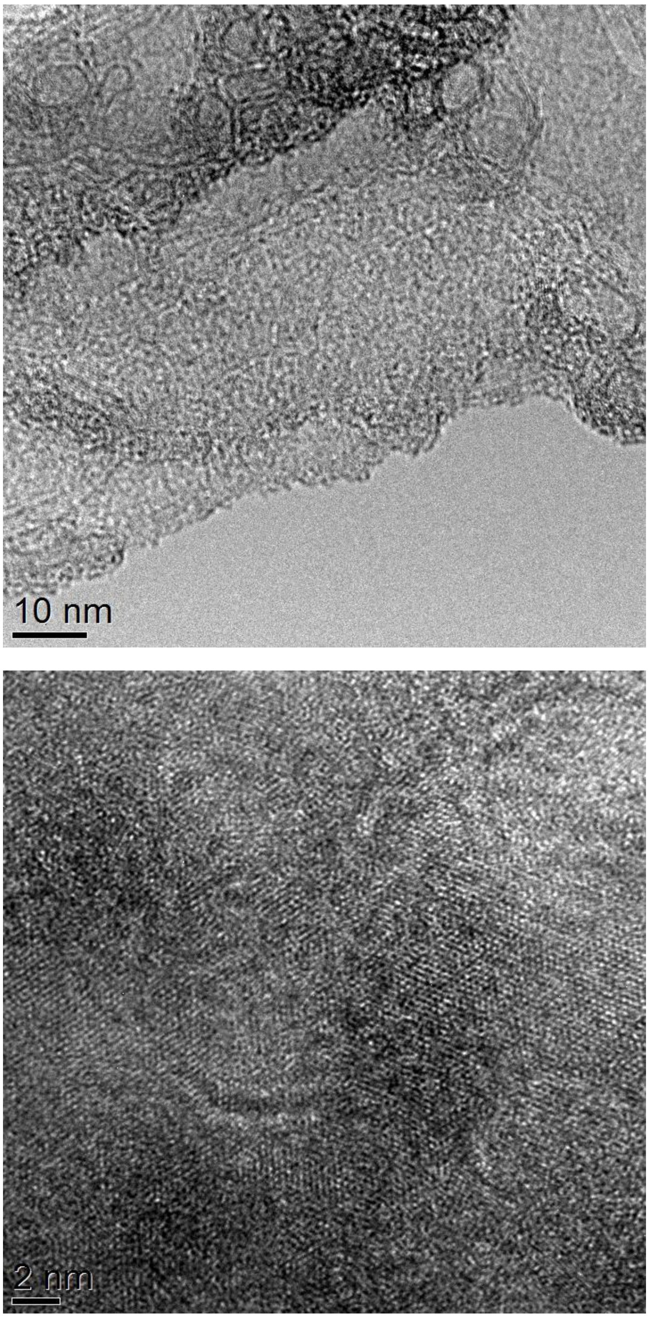

Supplementary Figure 5 | HRTEM analysis of PTC-Fe at different magnification.

High resolution TEM image shows poly-crystalline, honeycomb-like networks.

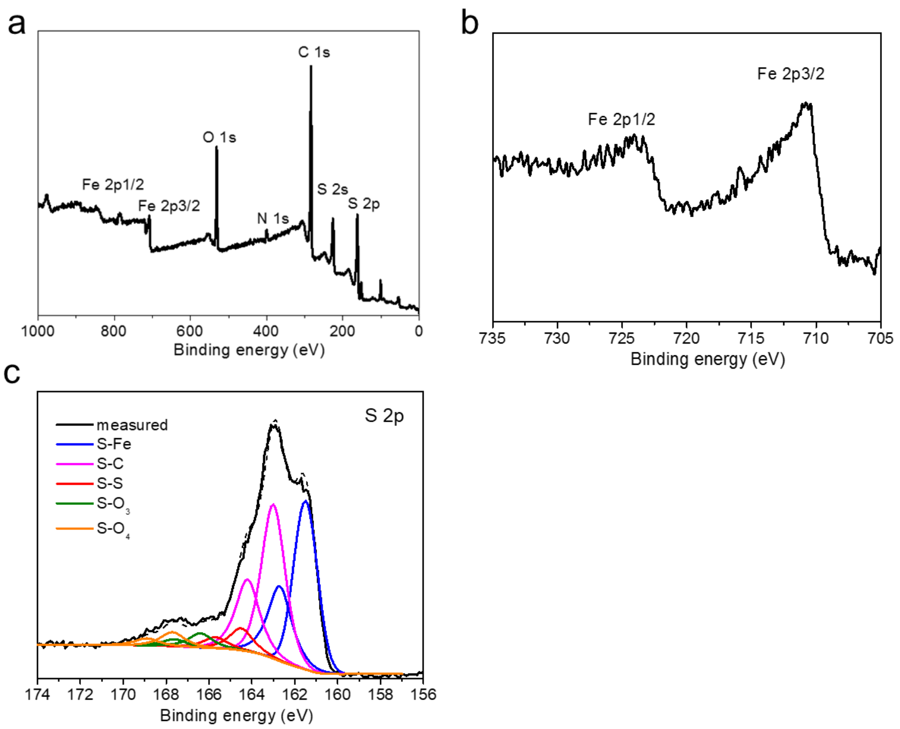

Supplementary Figure 6 | XPS analysis of PTC-Fe 2D MOF. a, Energy survey spectrum. **b**, high-resolution spectrum in the Fe 2p region. **c**, high-resolution spectrum in the S 2p region. The doublet peaks with an intensity ratio of 1:2 are due to spin orbit coupling, with Δ=1.2 eV, and are characteristic of the S 2p3/2 and 2p1/2 orbitals. The high-intensity dual peaks at 161.5 and 162.7 eV derive from the -Fe-S- units while the major dual peaks at 163 and 164.2 eV indeed corresponding to the -C-S- units. The weak peaks at 164.5 and 165.7 eV are assigned to a negligible fraction of -S-S- bonds.

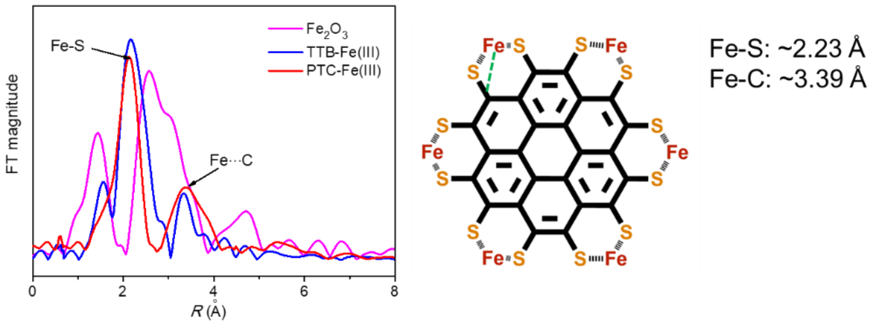

Supplementary Figure 7 | Fourier transform of the EXAFS at Fe K-edge of synthesized PTC-Fe MOF as well as Fe_2O_3 and TTB-Fe as the contrast samples.

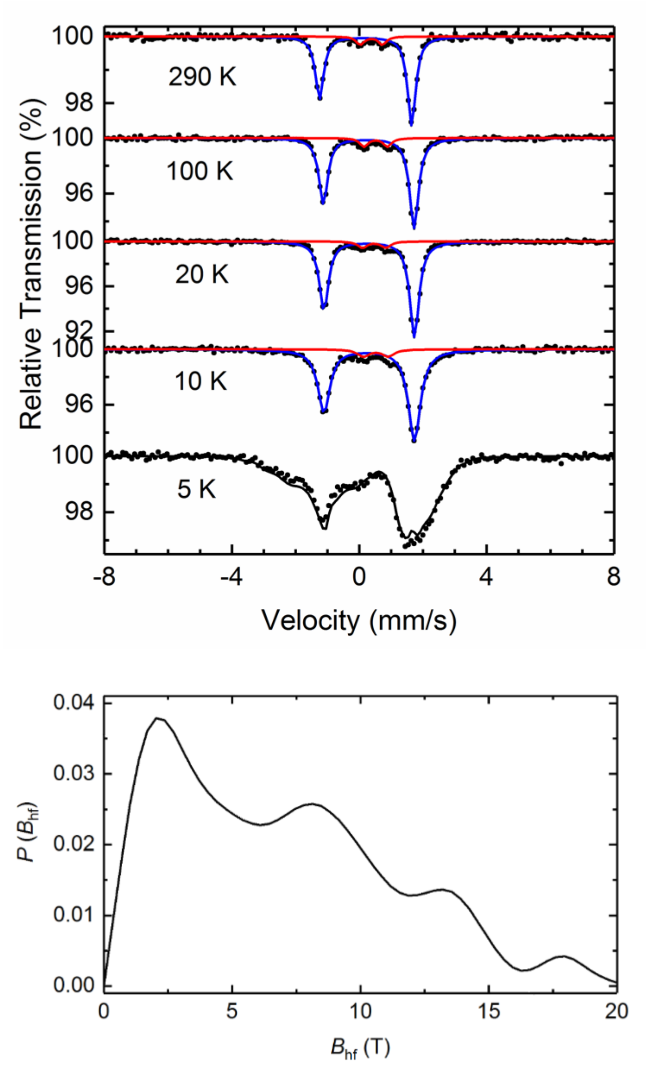

Supplementary Figure 8 | ^{57}Fe Mössbauer spectra of PTC-Fe at the indicated temperatures. Spectra between 290 and 10 K feature a quadrupole doublet (blue) for PTC-Fe which verifies a paramagnetic state in this temperature range. The asymmetry reflects a texture effect. The small (red) doublet with a temperature independent area fraction was less pronounced in an initial measurement at room temperature (Fig. 2c) and is attributed to a deterioration product formed during storage prior to the temperature dependent measurements. The spectrum at 5 K features magnetic hyperfine splitting and was fitted by using the full Hamiltonian for combined electric quadrupole and magnetic hyperfine interaction and a broad hyperfine field distribution (bottom). Here, the texture effect is neglected which explains deviations between experimental and calculated spectra. Line broadening at 10 K signals the onset of spin freezing.

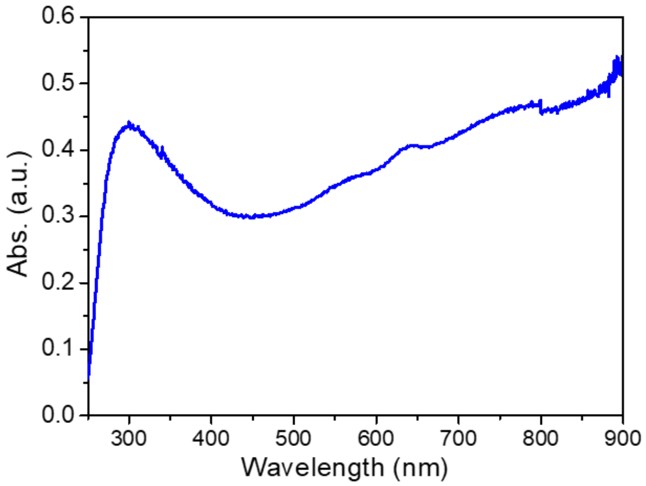

Supplementary Figure 9 | Solid-state UV-Vis absorption spectrum of PTC-Fe MOF. Importantly, the electronic absorption features of PTC-Fe MOF extend well into the near-infrared (NIR) range. Such low-energy electronic excitations are common in highly conjugated organic/metal-organic and conducting polymers.

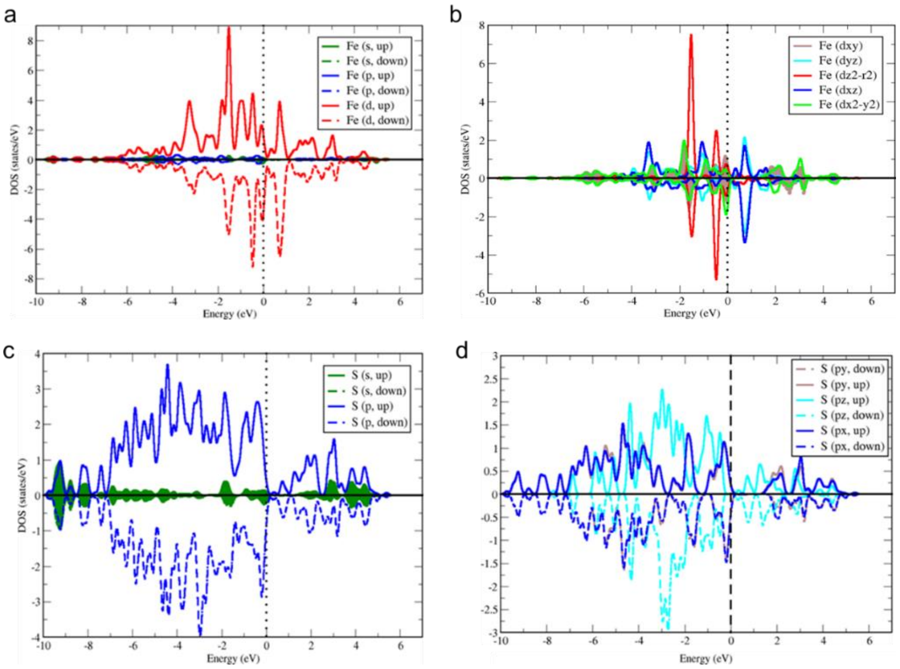

Supplementary Figure 10 | Band structure of single layer PTC-Fe. a and **b**, PDOS of Fe states. **c** and **d**, PDOS of S components.

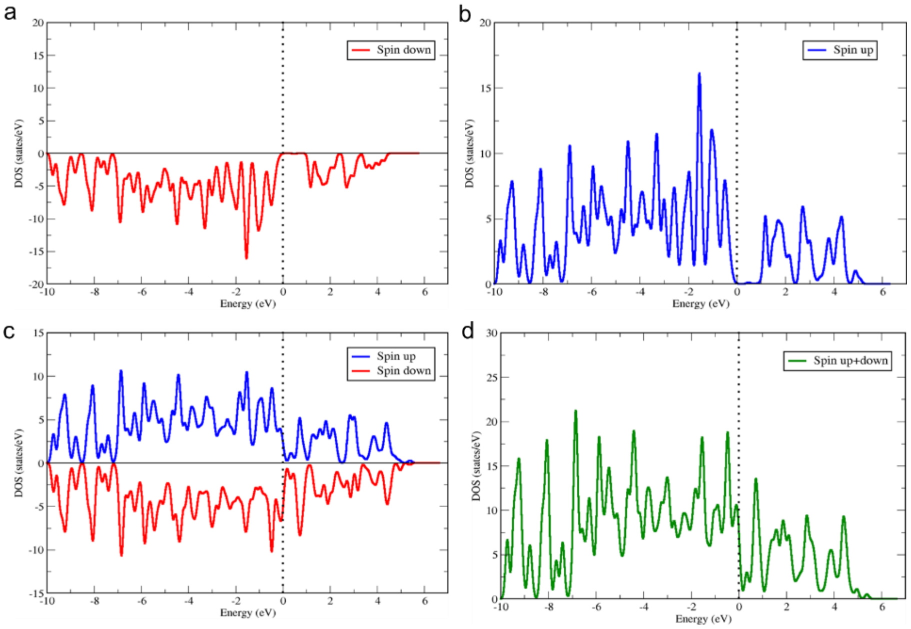

Supplementary Figure 11 | Band structure of single layer PTC-Fe. a, Band structure of PTC-Fe with the spin-down state of the Fe atoms. **b**, Band structure of PTC-Fe with the spin-up state of the Fe atoms. **c**, Band structure of PTC-Fe with the spin-down and spin-up states of the Fe atoms. **d**, Total DOS of the system (spin-up and spin down). Supplementary Fig. 11a presents only the spin-down state of the Fe atoms in single-layer system. It means that, in the input file for VASP, we have specified only spin-down calculations for Fe. In this case, the spin-down state reveals a band gap of ~1 eV for the Fe atoms. Similarly, Supplementary Fig. 11b only shows the spin-up state of the Fe, which also suggests a band gap of ~1 eV for the Fe spin-up system. While Supplementary Fig. 11c shows the spin-up and spin-down states of the Fe atoms, revealing a band gap of ~0.2 eV. The total DOS composed of the contribution from all atoms (Supplementary Fig. 11d), which displays a rather narrow band gap of ~0.2 eV for the single-layer system. It implies that the spin-up states for this system mainly present very close to the fermi level. Definitely, the calculation of a band gap for the whole system needs the contribution from both the spin-up and spin-down components.

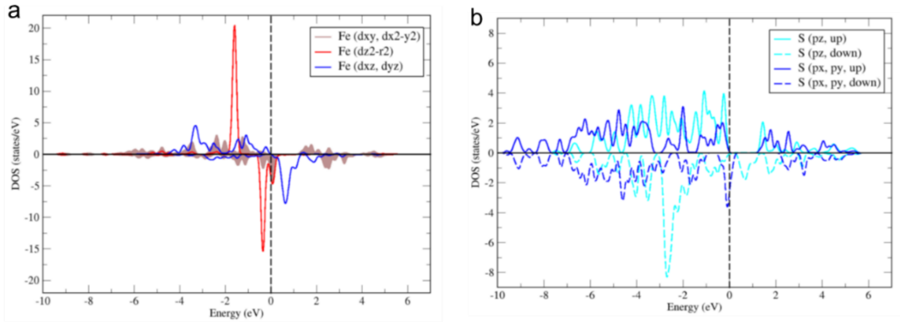

Supplementary Figure 12 | Band structure of multi-layer PTC-Fe with AB stacking model. a, PDOS of Fe states. **b**, PDOS of S components.

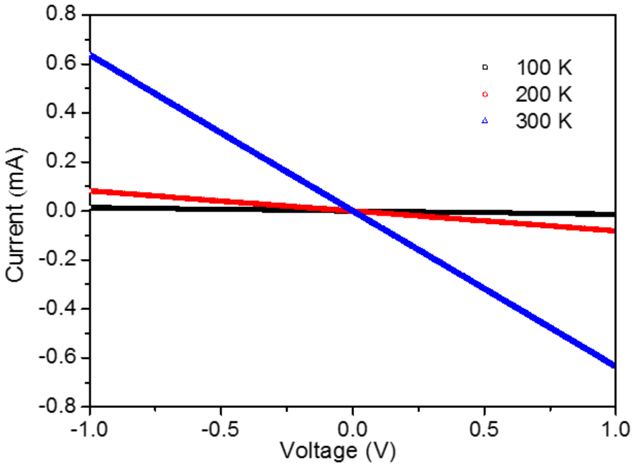

Supplementary Figure 13 | Typical variable temperature I-V curves of the PTC-Fe, displaying Ohmic response between –1.0 and 1.0 V.

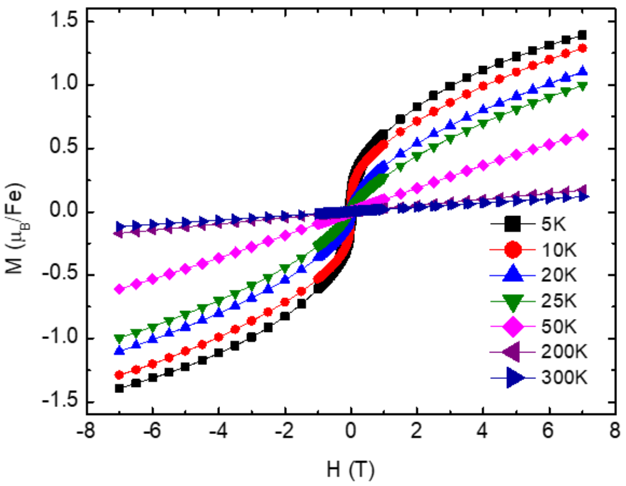

Supplementary Figure 14 | Magnetizations as functions of applied magnetic field (H) measured at different temperatures.

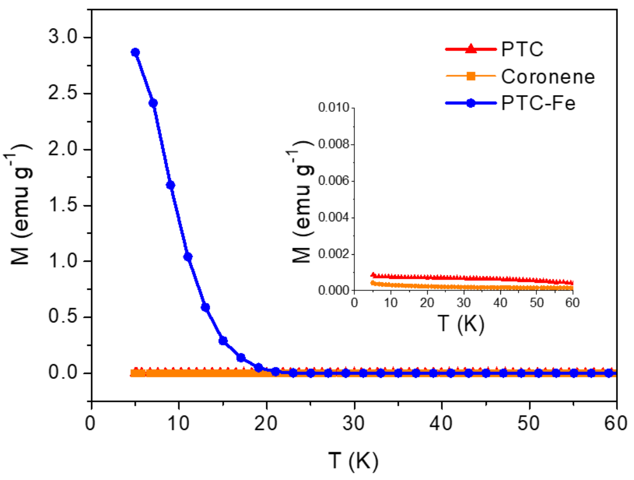

Supplementary Figure 15 | Temperature dependent remanent magnetization of ligand PTC (red curve), pristine coronene (yellow curve) and PTC-Fe MOF (blue curve). Inset: the enlarged image.

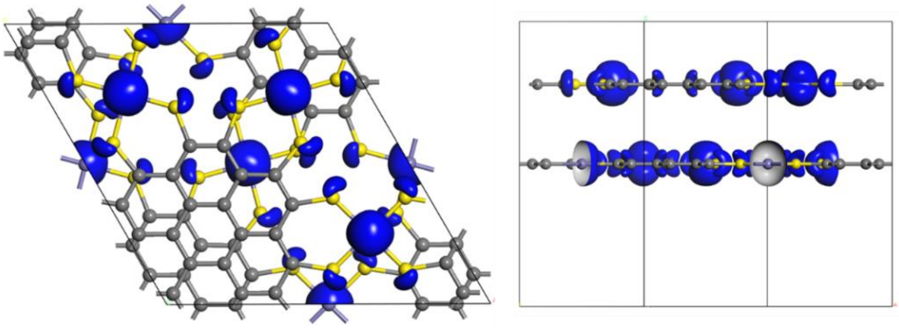

Supplementary Figure 16 | Spin density distribution of AB-stacking PTC-Fe MOF with 25% shifting in X and Y directions between neighboring layers.

Supplementary Tables

Supplementary Table 1 | Fitting parameters of the Mössbauer spectra of PTC-Fe. The spectra in Fig. S8 are described by the following parameters: isomer shift *IS*, quadrupole splitting *QS*, line width *Γ*, the magnetic hyperfine field B_{hf} (here average of the B_{hf} distribution), the asymmetry parameter η ($0 \leq \eta \leq 1$) of the electric field gradient (efg), the polar angle *Ω* describing the relative orientation between the principal component V_{ZZ} of the efg and B_{hf}. For the magnetic hyperfine pattern *QS* is obtained as $QS = eQV_{ZZ}/2(1 + \eta^2/3)^{1/2}$, where *Q* is the quadrupole moment of the excited ^{57}Fe nucleus. Note that $\eta = 0$ is in agreement with a square planar coordination and Ω ~ 90° indicates that the spins are oriented in the FeS_4 plane.

T (K)	*IS* (mm/s)	*QS* (mm/s)	*η*	B_{hf} (T)	Ω (°)	*Γ* (mm/s)	Area (%)
290	0.199(3)	2.890(5)				0.311(7)	91
	0.38(2)	0.70(4)				0.29(6)	9
200	0.250(2)	2.872(3)				0.330(4)	91
	0.48 (1)	0.77(2)				0.24(3)	9
100	0.296(2)	2.846(3)				0.339(4)	91
	0.52(2)	0.74(3)				0.30(3)	9
20	0.310(2)	2.839(3)				0.352(3)	93
	0.47(2)	0.73(3)				0.36(4)	7
10	0.311(2)	2.839(4)				0.463(6)	92
	0.52(3)	0.78(4)				0.46(6)	8
5.3	0.30(1)	+2.74(3)	0.0(1)	av. 6.3	93(2)	distr.	

Supplementary Table 2 | Currently reported 2D MOFs and their electrical conductivity values

Compound Formula	Organic Ligands	Metal ions	Conductivity at room temperature (S cm^{-1})	Ref.
**2D MOF powders by solvothermal synthesis**				
$Fe_3(PTC)$	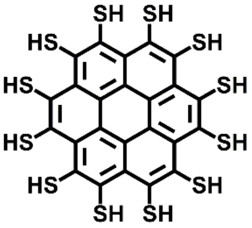	Fe^{3+}	~10 (Pellet, van der Pauw)	**This work**
$Ni_3(HTB)_2$	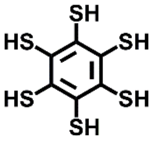	Ni^{2+}	0.15 (Pellet, 2-probe)	1
$Ni_3(HIB)_2$	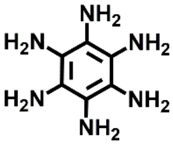	Ni^{2+}	8 (Pellet, van der Pauw)	2
$Cu_3(HIB)_2$	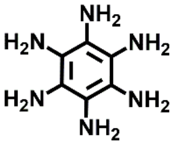	Cu^{2+}	13 (Pellet, van der Pauw)	2
$Cu_3(HHTP)_2$	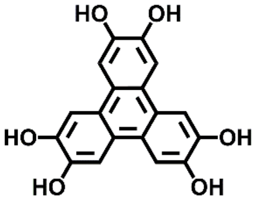	Cu^{2+}	0.2 (crystal, 4-probe)	3
$Ni_3(HITP)_2$	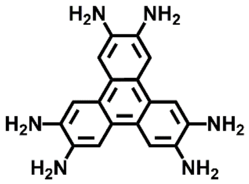	Ni^{2+}	2 (Pellet, 2-probe)	4
$Cu_3(HITP)_2$	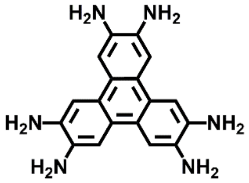	Cu^{2+}	0.2 (Pellet, 2-probe)	5

$Co_3(HTTP)_2$	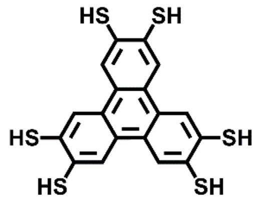	Co^{2+}	0.001 (Pellet, van der Pauw)	6
$Pt_3(HTTP)_2$	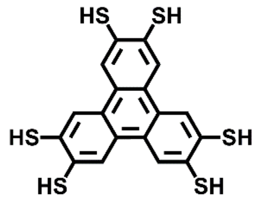	Pt^{2+}	3.8×10^{-6} (Pellet, 2-probe)	7
**2D MOF films by interfacial synthesis**				
$Ni_3(HTB)_2$	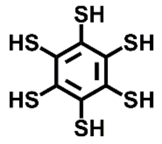	Ni^{2+}	160 (Film, van der Pauw)	8
$Cu_3(HTB)$	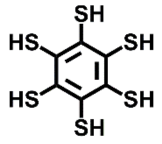	Cu^{2+}	1580 (Film, 4-probe)	9
$Ni_3(ITB)_2$	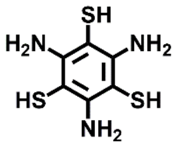	Ni^{2+}	0.1 (Film, van der Pauw)	10
$M_3(HIB)_2$	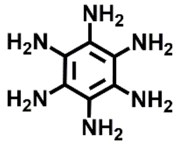	M= Co^{2+} Ni^{2+}, Cu^{2+}	Low conductivity (Film, van der Pauw)	11
$Ni_3(HITP)_2$	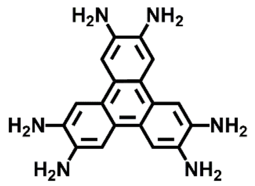	Ni^{2+}	40 (Film, van der Pauw)	4
$Co_3(HTTP)_2$	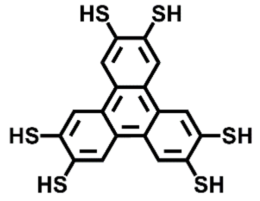	Co^{2+}	0.032 (Film, van der Pauw)	7
$Fe_3(HTTP)_2$	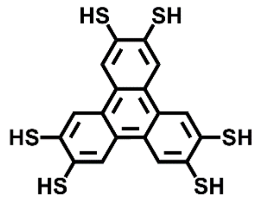	Fe^{3+}	1.1 (Film, 2-probe)	12

Supplementary Methods

Materials

Starting materials (e.g., e.g., $AlCl_3$, ICl, CCl_4, coronene, benzyl mercaptan, lithium, sodium hydride, 1,3-dimethyl-2-imidazolidinone (DMI), $NaBH_4$, $Fe(OAc)_2$) were purchased from Sigma-Aldrich. Liquid ammonium (purity >99.999 Vol.%) was purchased from Air Liquid GmbH (Germany). Unless otherwise stated, the commercially available reagents and dry solvents were used without further purification. Water was purified using a Milli-Q purification system (Merck KGaA). The reactions were performed using standard vacuum-line and Schlenk techniques. Work-up and purification of all compounds were performed in air and with reagent-grade solvents. Column chromatography was performed with silica gel (particle size 0.063-0.200 mm; obtained from Macherey-Nagel), and silica-coated aluminum sheets with a fluorescence indicator (obtained from Macherey-Nagel) were used for thin-layer chromatography.

The ligands, 1,2,3,4,5,6,7,8,9,10,11,12-pertiolated coronene (PTC)[13] and 1,2,4,5-tetrathiolbenzene (TTB)[14], were synthesized following the reported protocols, respectively. The TTB-Fe(III) coordination polymer was synthesized according to our previous method[15].

General characterization

UV-visible spectra were measured on a Cary 5000 UV-Vis-NIR (Agilent Technologies) spectrophotometer at room temperature using a 10-mm quartz cell and a 3 cm * 3 cm quartz wafer. Infrared spectra were recorded on a FT-IR Spectrometer Tensor II (Bruker) with an ATR unit. ^{1}H NMR and ^{13}C NMR spectra for the synthesis of the ligands were recorded in deuterated solvents on a Bruker DPX 250 spectrometer. High resolution MALDI-TOF mass spectra were recorded on a Bruker Reflex II-TOF spectrometer using a 337-nm nitrogen laser with TCNQ as the matrix.

The morphology and structure of the samples were investigated by transmission electron microscopy (TEM, Carl Zeiss Libra 200 MC Cs), scanning electron

microscopy (SEM, Carl Zeiss Gemini 500), and optical microscopy (Zeiss) with a Hitachi KP-D50 color digital CCD camera. Energy dispersed X-ray spectroscopy (EDS) was performed using a monochromatic Al Kα radiation source (1486.6 eV). X-ray powder diffraction (XRD) was carried out on Siemens D5000 X-ray diffractometer using Co Kα (1.79 Å) radiation at room temperature. X-ray photoelectron spectroscopy (XPS) measurements were carried out using an AXIS Ultra DLD system from Kratos with Al Kα radiation. Both survey and high-resolution spectra were collected using a beam diameter of 100 μm. The instrument was calibrated following the ISO 15472 protocol, and spectra were referred to the Au47/2 peak at 84 eV. The spectra were processed with CasaXPS software (version 2.3.15, Casa Software Ltd, Wilmslow, Cheshire, UK). Nitrogen sorption measurements were conducted at 77 K on a Quantachrome volumetric analyser. All samples were degassed at 100 ºC for at least 4 h before every measurement. Specific surface areas were determined by the standard BET method based on the relative pressure between 0.05 and 0.20.

<u>^{57}Fe Mössbauer spectroscopy measurements</u>

Mössbauer spectra of PTC-Fe were collected between 5 and 294 K with a standard WissEl spectrometer, which was operated in the constant acceleration mode and which was equipped with a ^{57}Co/Rh source. The sample consisted of thin sheets of PTC-Fe which could not be properly ground to a powder. Accordingly, texture effects are apparent in the spectra. About 30 mg of sample was filled into a Plexiglass sample container with inner diamter of 13 mm. Spectra were obtained at various temperatures using a Janis-SHI-850-5 closed cycle refrigerator (CCR). The isomer shifts are given relative to α-iron. The data were evaluated with the program MossWinn[16] within the thin absorber approximation. Spectra in the paramagnetic phase were described by doublets with Lorentzian line shapes where the intensity ratio between the two doublets was allowed to vary in order to account for the texture effects. The low temperature spectrum ($T \sim 5$ K) was evaluated by diagonalizing the full Hamiltonian for combined electric quadrupole and magnetic hyperfine interaction and by assuming a hyperfine field distribution which was extracted using the Hesse-Rübartsch method implemented

in MossWinn. The evaluation of the 5 K spectrum does not take into account the texture effects.

XAS measurements

All X-ray Absorption Fine Structure (XAFS) data were measured at the beamline BL14W1 in Shanghai Synchrotron Radiation Facility (SSRF, China) which was operated at the top-up mode with maximum current of 260 mA with a Si(111) double crystal monochromator. A N_2-filled ionization chamber was used to measure the incident flux. Data of the PTC-Fe and TTB-Fe(III) were collected in the transmission mode diluted with LiF to reduce thickness effects. The energy was calibrated using Fe foil. The size of the Synchrotron beam at the sample location was 0.3 mm(V)×0.3 mm(H). During the XAFS measurement, samples were maintained at room temperature. Multiple scans were measured and averaged. The replicate spectra were reproducible indicating there was no measurable impact of beam damage.

Modeling and electronic structure of PTC-Fe

For the periodic structures DFT calculations were performed using the program VASP (Vienna Ab Initio Simulation Package)[17-20] where the electronic wave functions have been expanded into plane waves up to an energy cutoff of 400 eV and a projected-augumented-wave (PAW)[21] scheme has been used to describe the interactions between the valence electrons and the nuclei (ions). The exchange correlation interactions between electrons were treated within the generalized gradient approximation (GGA) as implemented by Perdew, Burke and Ernzerhof (PBE).[22] This code projects the VASP Kohn-Sham wave functions onto atomic Bader volumes and calculates the corresponding density of states (DOS) within these volumes. Besides DOS, the PDOS (projected density of states) has been also calculated. PDOS is calculated as projected DOS, where the information about the different contributions of the different orbitals is computed.

The minimum energy configurations were considered to be converged when the forces on each atom of the molecules were less than 0.02 eV/Å.

Two different type of stacking have been investigated: single layer and AB.

For the AB stacking the following possibilities have been taken into account:

- the B layer has been shifted with half unit cell (50%) on x direction and half unit cell (50%) on y direction compared with A layer.
- the B layer has been only shifted with 50% on x direction compared with A layer
- the B layer has been only shifted with 50% on y direction compared with A layer
- the B layer has been shifted only with 25% on x direction compared with A layer
- the B layer has been shifted only with 25% on y direction compared with A layer
- the B layer has been shifted both in x and y direction with 25% compared with A layer

Simulation of Curie temperature

In order to estimate the magnetic exchange interactions, we employed the Ising model[23,24] $E_{tot} = \sum_{ij} J_{ij} \vec{S_i} \cdot \vec{S_j}$, with various spin configurations, where E_{tot} is the total energy obtained from DFT calculations, J_{ij} is the exchange interaction between the *i*th and *j*th sites and $\vec{S_i}$ and $\vec{S_j}$ are the effective spin values at the *i*th and *j*th sites, respectively.

We considered the nearest (J_1) and next nearest neighbor (J_2) approximations. The representative spin configurations and the corresponding energy expression:

$E_{FM} = 36J_1 + 36J_2$

$E_{AFM1} = -36J_1 + 36J_2$

$E_{AFM2} = 36J_1 - 36J_2$

$J_1 = 2.7\ meV$

$J_2 = 2.7\ meV$

The curie temperature is

$$T_c = \frac{(J_1 + J_2)S(S+1)}{3k_B} = 16\ K$$

Where, $S(S+1)$=3/4, k_B is the Boltzmann constant.

Supplementary References

1. Kambe, T. et al. π-Conjugated nickel bis(dithiolene) complex nanosheet. *J. Am. Chem. Soc.* **135**, 2462-2465 (2013).
2. Dou, J.-H. et al. Signature of metallic behavior in the metal-organic frameworks M3(hexaiminobenzene)2 (M = Ni, Cu). *J. Am. Chem. Soc.* **139**, 13608-13611 (2017).
3. Hmadeh, M. et al. New porous crystals of extended metal-catecholates. *Chem. Mater.* **24**, 3511-3513 (2012).
4. Sheberla, D. et al. High electrical conductivity in Ni_3(2,3,6,7,10,11-hexaiminotriphenylene)$_2$, a semiconducting metal-organic graphene analogue. *J. Am. Chem. Soc.* **136**, 8859-8862 (2014).
5. Campbell, M.G. et al. Cu_3(hexaiminotriphenylene)$_2$: An Electrically Conductive 2D Metal-Organic Framework for Chemiresistive Sensing. *Angew. Chem. Int. Ed.* **54**, 4349-4352 (2015).
6. Clough, A.J. et al. Metallic conductivity in a two-dimensional cobalt dithiolene metal-organic framework. *J. Am. Chem. Soc.* **139**, 10863-10867 (2017).
7. Cui, J. & Xu. Z. An electroactive porous network from covalent metal–dithiolene links. *Chem. Commun.* **50**, 3986-3988 (2014).
8. Kambe, T. et al. Redox control and high conductivity of nickel bis(dithiolene) complex π-nanosheet: a potential organic two-dimensional topological insulator. *J. Am. Chem. Soc.* **136**, 14357-14360 (2014).
9. Huang, X. et al. A two-dimensional π-d conjugated coordination polymer with extremely high electrical conductivity and ambipolar transport behaviour. *Nat. Commun.* **6**, 7408 (2015).
10. Sun, X. et al. Conducting π-conjugated bis(iminothiolato)nickel nanosheet. *Chem. Lett.* **46**, 1072-1075 (2017).
11. Lahiri, N. et al. Hexaaminobenzene as a building block for a family of 2D coordination polymers. *J. Am. Chem. Soc.* **139**, 19-22 (2017).

12. Dong, R. et al. Large-area, free-standing, two-dimensional supramolecular polymer single-layer sheets for highly efficient electrocatalytic hydrogen evolution. *Angew. Chem. Int. Ed.* **54**, 12058-12063 (2015).
13. Dong, R. et al. Persulfurated coronene: a new generation of “sulflower”. *J. Am. Chem. Soc.* **139**, 2168-2171 (2017).
14. Dirk, C. W., Cox, S. D., Wellman, D. E. & Wudl, F. Isolation and purification of benzene-1, 2, 4, 5-tetrathiol. *J. Org. Chem.* **50**, 2395 (1985).
15. Wang, L. et al., Toward activity origin of electrocatalytic hydrogen evolution reaction on carbon-rich crystalline coordination polymers. *Small* **13**, 1700783 (2017).
16. Klencsár, Z.; Kuzmann, A.; Vértes, A. User-Friendly Software for Mössbauer Spectrum Analysis. *J. Radioanal. Nucl. Chem.* **210,** 105−118 (1996).
17. Kresse, G. & Hafner, J. Ab initio molecular dynamics for liquid metals. *Phys. Rev. B* **47**, 558-561 (1993).
18. Kresse, G. & Furthmuller, J. Efficiency of Ab-initio total energy calculations for metals and semiconductors using a plane-wave basis set. *Computat. Mater. Sci.* **6**, 15-50 (1996).
19. Kresse, G. & Furthmuller, J. Efficient iterative schemes for Ab initio total-energy calculations using a plane-wave basis set. *Phys. Rev. B* **54**, 11169-11186 (1996).
20. Kresse, G. & Joubert, D. From ultrasoft pseudopotentials to the projector augmented-wave method. *Phys. Rev. B* **59**, 1758-1775 (1999).
21. Blochl, P. E. Projector augmented-wave method. *Phys. Rev. B* **50**, 17953-17979 (1994).
22. Perdew, J. P.; Burke, K.; Ernzerhof, M. Generalized gradient approximation made simple. *Phys. Rev. Lett.* **77**, 3865-3868 (1996).
23. Li, W. et al. High temperature ferromagnetism in π-conjugated two-dimensional metal-organic frameworks. *Chem. Sci.* **8**, 2859-2867 (2017).
24. Zhou, J. & Sun, Q. Magnetism of phthalocyanine-based organometallic single porous sheet. *J. Am. Chem. Soc.* **133**, 15113-15119 (2011).
